# *N*-Heterocyclic carbene/Brønsted acid cooperative catalysis as a powerful tool in organic synthesis

**DOI:** 10.3762/bjoc.8.43

**Published:** 2012-03-14

**Authors:** Rob De Vreese, Matthias D’hooghe

**Affiliations:** 1 SynBioC Research Group, Department of Sustainable Organic Chemistry and Technology, Faculty of Bioscience Engineering, Ghent University, Coupure links 653, B-9000 Ghent, Belgium

**Keywords:** Brønsted acids, cooperative catalysis, γ-lactams, *N*-heterocyclic carbenes, stereoselectivity

## Abstract

The interplay between metals and *N*-heterocyclic carbenes (NHCs) has provided a window of opportunities for the development of novel catalytic strategies within the past few years. The recent successful combination of Brønsted acids with NHCs has added a new dimension to the field of cooperative catalysis, enabling the stereoselective synthesis of functionalized pyrrolidin-2-ones as valuable scaffolds in heterocyclic chemistry. This Commentary will briefly highlight the concept of *N*-heterocyclic carbene/Brønsted acid cooperative catalysis as a new and powerful methodology in organic chemistry.

## Introduction

**Scheme 1:**
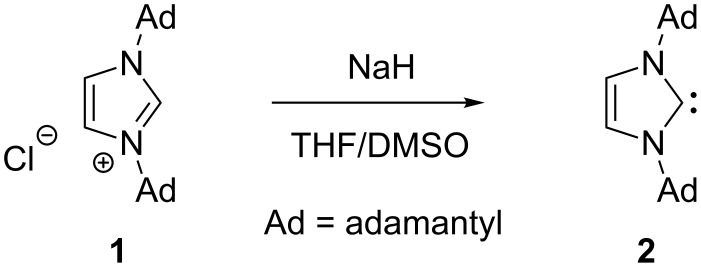
Synthesis of the first free and stable *N*-heterocyclic carbene by Arduengo [2].

Carbenes have been the topic of intensive research for more than 150 years, and they continue to attract considerable attention from chemists to date. Whereas many attempts to isolate methylene or related compounds failed, Fischer provided the first preparation and characterization of a metal carbene complex in 1964 through nucleophilic attack of phenyllithium at tungsten hexacarbonyl followed by *O*-alkylation [1], and Arduengo described the preparation of the first free and stable *N*-heterocyclic carbene **2** by deprotonation of the corresponding imidazolium salt **1** in 1991 (Scheme 1) [2]. Two years earlier, Bertrand had reported the synthesis of [bis(diisopropylamino)phosphino]trimethylsilylcarbene as the first isolated free carbene [3]. The isolation of stable NHCs and their successful applications as ligands for the preparation of various metal complexes encouraged many chemists to search intensively for new NHC ligands, and this has led to the establishment of a very fruitful research area in organic chemistry [4–10].

**Scheme 2:**
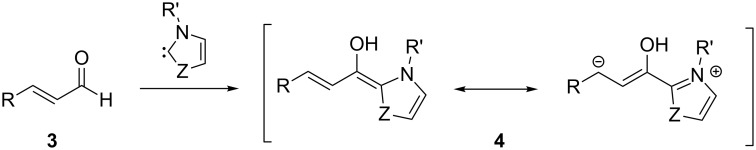
Conjugate “umpolung” of α,β-unsaturated aldehydes.

A particularly interesting application comprises the use of “umpolung” reactions [11] (inversion of polarity) catalyzed by *N*-heterocyclic carbenes, such as the benzoin condensation and the Stetter reaction. In these reactions, the NHC effects an “umpolung” of the normal carbonyl reactivity, and the electrophilic aldehyde carbon atom thus becomes nucleophilic and can attack a variety of electrophiles. The story of “umpolung” reactions of aldehydes started as long ago as 1832, and the preparation of sterically hindered triazolium salts in 1996 provided a solid basis for highly stereoselective “umpolung” reactions utilizing NHCs [12]. In addition, conjugate “umpolung” relates to a process in which α,β-unsaturated aldehydes **3** are transformed into d^3^-nucleophiles or homoenolate equivalents **4** through addition of a nucleophilic catalyst across the aldehyde functionality (Scheme 2), a peculiar feature that has been used for the straightforward synthesis of γ-butyrolactones through reaction with aromatic aldehydes [13–14].

In recent years, the concept of cooperative catalysis has emerged as a powerful technique for the highly selective synthesis of a variety of target structures. Although carbenes can act as ligands for metals, thus inhibiting the individual reactivity of each component, recent studies have shown that in some cases combinations of metals (Lewis acids) and carbenes can be used as a novel catalytic system [15–17]. Moreover, this type of cooperation has been demonstrated to feature a unique reactivity that is difficult to achieve by using one of both catalysts individually. The main challenge in that respect involves the search for suitable combinations and, if one of both partners is chiral, the development of enantioselective catalytic processes. Selected examples of the successful deployment of cooperative catalysis in organic synthesis comprise the preparation of chiral γ-lactams from *N*-acyl hydrazones and α,β-unsaturated aldehydes [18], the enantioselective synthesis of cyclopentenes from α,β-unsaturated aldehydes and α,β-unsaturated ketones [19], and the preparation of cyclopentanes through the reaction of enals and β,γ-unsaturated α-ketoesters [20].

## Discussion

**Scheme 3:**
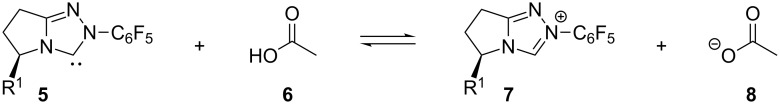
The carbene + conjugate acid – azolium + base equilibrium.

Recently, Rovis et al. reported that the acetate anion **8** can deprotonate pentafluorophenyl triazolium salts **7** to give the free carbenes **5** and acetic acid **6**, pointing to the peculiar conclusion that this carboxylic acid does not neutralize the carbenes (Scheme 3) [21]. Probably, the presence of the electron-withdrawing pentafluorophenyl group in carbene precursors **7** has an important effect on the basicity and thus on the overall reactivity. In this way, the NHC and the conjugate acid can be present in sufficient quantities to promote a new form of cooperative catalysis. Further elaboration of this interesting observation led to the hypothesis that the combination of a pentafluorophenyl triazolium carbene **5** and a Brønsted acid with low p*K*_a_ value may provide new opportunities for the design of reaction pathways in which the carbene and the acid play different roles. The idea to use a very weak base in NHC catalysis has also been described by Bode et al., who showed that there is no need to add a base when employing azolium catalysts with more basic counterions [22].

**Scheme 4:**
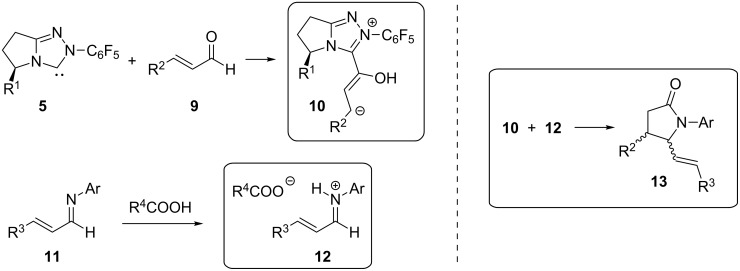
Formation of Breslow intermediates **10** and iminium salts **12** and their use toward the synthesis of γ-lactams **13**.

As mentioned in the introduction, the reaction of NHCs with enals is known to produce homoenolates [13–14], which have found useful applications as vinylogous acyl anions toward the synthesis of different heterocycles. Nonetheless, enantioselective control of homoenolates remained an important issue to be addressed, and this problem was tackled very recently by the group of T. Rovis [23]. Their important contribution to the field of organocatalysis originated from the hypothesis that the conjugate acid of the base used to generate a carbene could (partially) convert an imine **11** to the corresponding highly electrophilic iminium salt **12,** while the deployment of a chiral carbene **5** could lead to a means of controlling the stereochemistry during the reaction of the in situ formed homoenolate **10** with the activated iminium salt **12** to provide a new entry into the class of pyrrolidin-2-ones **13** (Scheme 4), which is potentially complementary to other methods in terms of stereochemistry. However, the formation of lactones instead of lactams through the intervention of aldehydes as electrophiles could not be excluded in advance [16].

**Scheme 5:**
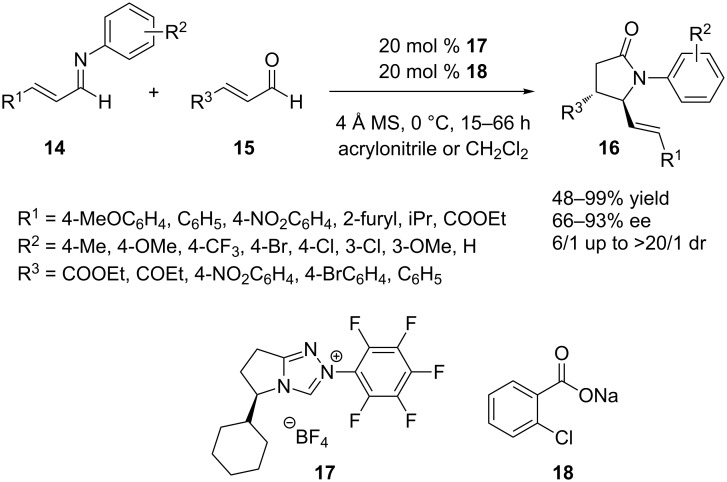
Synthesis of *trans*-γ-lactams **16** through NHC/Brønsted acid cooperative catalysis.

Application of this methodology indeed enabled the facile and stereoselective synthesis of *trans*-γ-lactams **16** in a straightforward manner (Scheme 5), which would not have been possible without cooperative catalysis. In a first attempt, this reaction was performed by using a rather strong base (KHMDS, Et_3_N) to deprotonate the carbene precursor, which led to the formation of a weak Brønsted acid with low yields and poor selectivities as a consequence. The use of weaker bases (carboxylates) resulted in stronger conjugate acids capable of activating imines **14**, thus affording better overall yields. It should be noted that electron-rich carbenes have historically been used to catalyze homoenolate chemistry rather than electron-poor catalysts such as **5**. Optimization of the temperature and the solvent led to an improved stereoselectivity, and variation of the used acid led to improved yields. Finally, the most efficient *trans*-γ-lactam synthesis has been achieved using cyclohexyl-substituted carbene precursor **17** (20 mol %) and sodium *o*-chlorobenzoate (**18,** 20 mol %) in the presence of molecular sieves at 0 °C and with acrylonitrile as solvent (although in some cases CH_2_Cl_2_ gave better results). Under these optimized conditions, the scope of the catalytic synthesis of *trans*-γ-lactams **16** was further investigated through the use of a variety of aldimines **14** with different substitution patterns (R^1^ and R^2^). In every case, the product **16** was shown to be formed in good yield and high diastereo- and enantioselectivity. A number of different enals **15** as suitable substrates has been explored as well (variation of R^3^), affording the *trans*-γ-lactam products **16** in moderate to good yields and stereoselectivities. It is important to underline the fact that this approach provides an unprecedented *trans* selectivity in the synthesis of 4,5-disubstituted γ-lactam systems.

**Figure 1:**
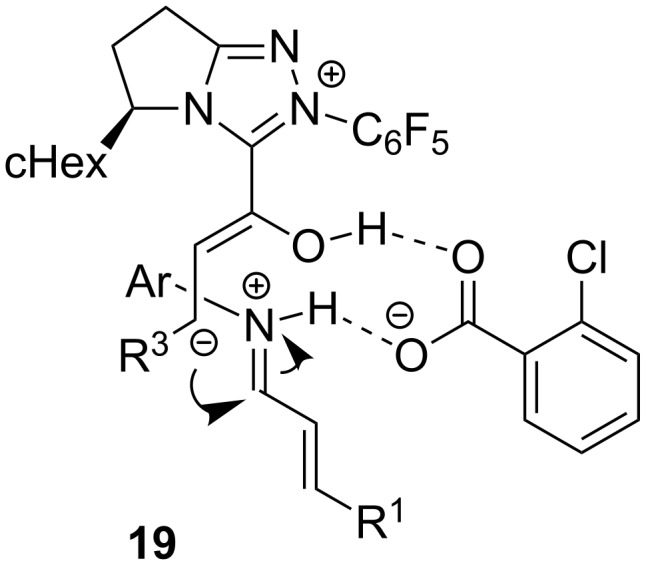
Proposed hydrogen-bonding intermediates **19** in the formation of pyrrolidin-2-ones **16**.

The occurrence of hydrogen-bonding intermediates **19** (Figure 1) has been invoked to explain the reaction mechanism, and the phenomenon of hydrogen bonding in NHC catalysis has indeed been described before to account for improved stereoselectivities [24–26].

Although the concept of cooperative catalysis involving NHCs had emerged previously in organic chemistry and the compatibility of NHCs and Brønsted acids had been reported by Rovis in 2010 [27], the NHC/Brønsted acid cooperative catalytic system had not been described before. This recent work of Rovis clearly puts the concept of cooperative catalysis in a new perspective, showing that the use of Brønsted acids in combination with NHCs provides a powerful approach toward the selective synthesis of well-defined targets.

Because of their interesting biological activities, their widespread occurrence in many natural products [28], and their broad synthetic utility, γ-lactam ring systems have received considerable attention in organic chemistry. Recent strategies toward the construction of the γ-lactam motif comprise β-lactam to γ-lactam ring expansions [29–30], aziridine ring openings followed by cyclization with enolates [31], palladium-catalyzed cyclizations [32], cycloadditions [33], multicomponent reactions [34], and even NHC catalysis [35–36]. Nevertheless, the methodology developed by Rovis indisputably represents an important breakthrough, providing an elegant alternative access to *trans*-γ-lactams in a highly enantio- and diastereoselective way.

Further elaboration and refinement of this methodology will certainly lead to a plethora of useful applications in the future. An issue to be addressed concerns the improvement of the efficiency of this catalytic process by lowering the catalyst load (20 mol % is too much). Another minor issue could relate to a limitation of the scope concerning the imines used in this work. Although the *N*-arylimine substrates **14** have been described as “unactivated” (which is true as compared to, for example, *N*-sulfonylimines), *N*-arylimines are more reactive as compared to *N*-alkylimines. The question of whether or not *N*-alkylimines can be deployed successfully in this methodology deserves further elaboration, and may have certain implications with regard to the bioactivity of the corresponding *N*-alkylpyrrolidin-2-ones due to a more basic nitrogen atom. In addition, it would be of interest to evaluate the preparation of NH γ-lactams, for example through oxidative removal of a 4-methoxyphenyl group, which in turn may be valuable scaffolds in medicinal chemistry. Also the necessity of α,β-unsaturated imines as reaction partners has neither been evaluated nor discussed, and *N*-(arylmethylidene)amines and *N*-(alkylidene)amines should be evaluated as alternative imine substrates. Finally, further study of preliminary results regarding the use of achiral carbenes in combination with chiral (amino) acids could deliver functionalized γ-lactams with high enantioselectivity as well.

## Conclusion

In summary, the use of the *N*-heterocyclic carbene/Brønsted acid system should be considered as a novel aspect of cooperative catalysis, providing new opportunities for research in this area. It is evident that further elaboration of this concept of cooperative catalysis holds promising prospects for asymmetric syntheses of valuable heterocyclic entities in a sustainable and elegant way.
